# P-1930. Absence of Booster Vaccination and Low Cycle Threshold Values Predict Severe and Critical COVID-19 in Fully Vaccinated Individuals: A Multi-Center Retrospective Cohort Study

**DOI:** 10.1093/ofid/ofae631.2090

**Published:** 2025-01-29

**Authors:** Charles Kevin L Rivera, Ia Marie Donna Cruz, Mark Carascal, Ma Cristina Perez, Raul V Destura, Karl Evans R Henson

**Affiliations:** The Medical City, Pasig City, National Capital Region, Philippines; Philippine General Hospital, Manila, National Capital Region, Philippines; The Medical City, Pasig City, National Capital Region, Philippines; The Medical City, Pasig City, National Capital Region, Philippines; The Medical City, Pasig City, National Capital Region, Philippines; The Medical City, Pasig City, National Capital Region, Philippines

## Abstract

**Background:**

The development of severe/critical Coronavirus Disease 2019 (COVID-19) infection in fully vaccinated individuals is an incompletely defined area of research and lacks clinical scoring tools. Most scoring systems were assessed with unvaccinated patients, making it less clinically applicable in today’s global landscape. Our study validated an existing clinical prediction tool using data from vaccinated individuals and created a new model that could provide improved prediction accuracy.

**Figure:**
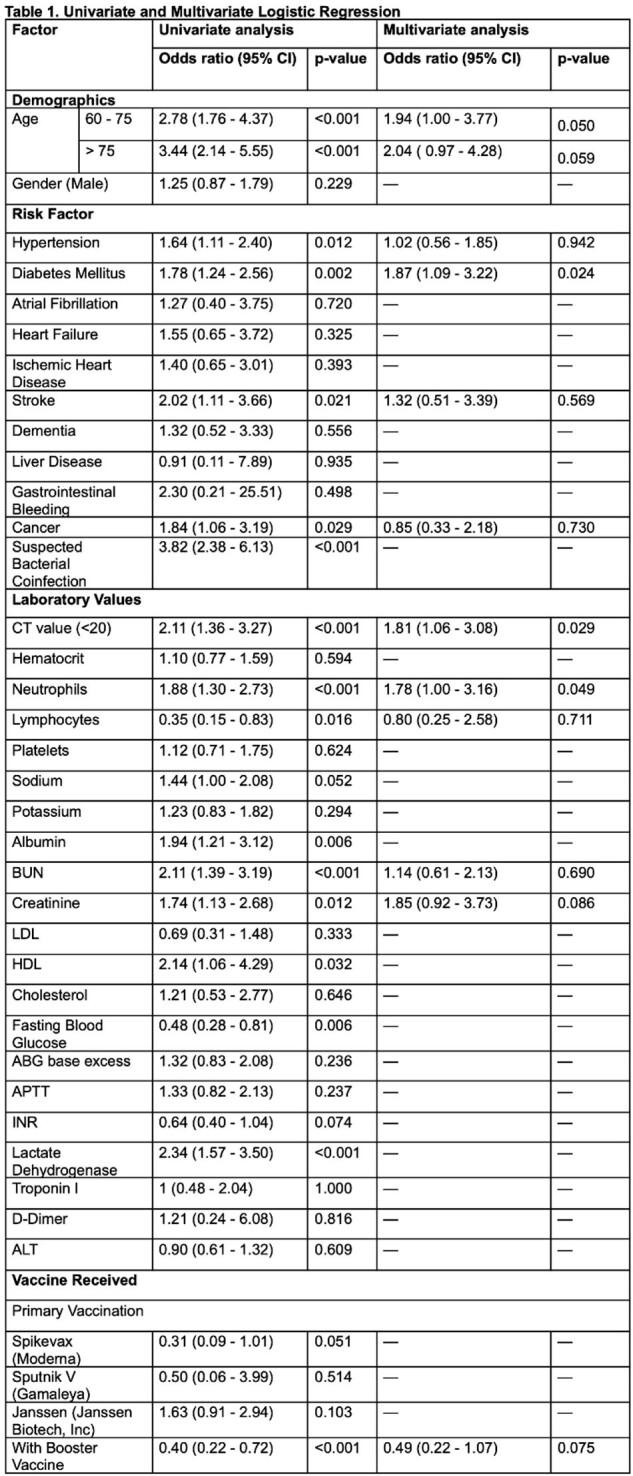

**Methods:**

Adults fully vaccinated against COVID-19 and admitted to two Philippine tertiary hospitals from March 2021 to August 2022 for breakthrough infections were enrolled. Clinical characteristics, type and number of vaccine doses, and rt-PCR cycle threshold (CT) values were collected. The primary outcome of interest was the development of severe/critical COVID-19. An existing severe/critical COVID-19 prediction tool was validated using the study data, while a new and simpler scoring system was developed from the multiple logistic regression analysis of significant variables.

**Table 2:**
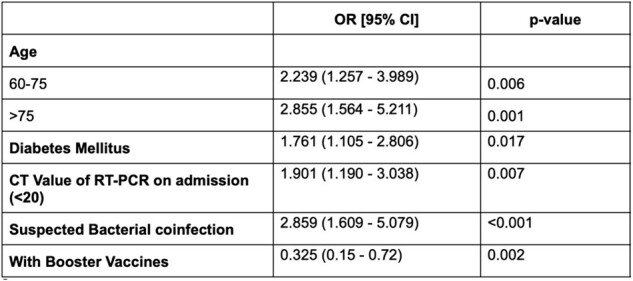
Final Model with Significant Factors

**Results:**

Among 803 adults with COVID-19, 138 (17%) received at least one booster dose, and 139 (17%) progressed to severe/critical disease. The existing prediction tool had a sensitivity (Sn) of 96.5%, specificity (Sp) of 11.7%, and area under the receiver-operator curve (AUROC) of 0.54. Multivariable logistic regression showed that age >75y (OR 2.86; 95% CI 1.56 - 5.21), diabetes mellitus (OR 1.76, 95% CI 1.10-2.80), CT value < 20 (OR 1.90; 95% CI: 1.19 - 3.03), suspicion of bacterial co-infection (OR 2.85, 95% CI 1.60-5.07), and non-receipt of a booster dose (OR 0.32; 95% CI: 0.15 - 0.72) were independent risk factors for developing severe/critical COVID-19. The developed scoring system showed a Sn of 89.4%, Sp of 62.7%, and AUROC of 0.76.

**Figure:**
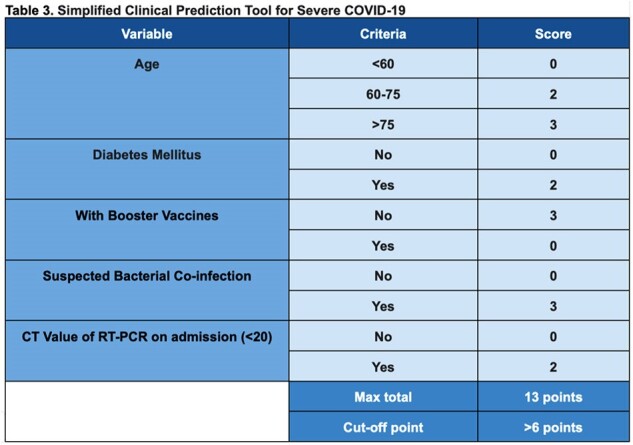

**Conclusion:**

A simplified scoring tool using five variables may help predict the progression to severe/critical COVID-19 in fully vaccinated individuals. Although the tool has modest Sn and Sp, it can help triage patients and guide initial therapy. Validation of this tool in larger studies is recommended.

**Disclosures:**

Karl Evans R. Henson, MD, FIDSA, BSV Biosciences: Honoraria|Cathay Drug Company, Inc: Grant/Research Support|MSD: Honoraria|Pfizer: Advisor/Consultant|Pfizer: Honoraria

